# Species-Specific Effects of Ant Inhabitants on Bromeliad Nutrition

**DOI:** 10.1371/journal.pone.0152113

**Published:** 2016-03-22

**Authors:** Ana Z. Gonçalves, Rafael S. Oliveira, Paulo S. Oliveira, Gustavo Q. Romero

**Affiliations:** 1 Pós-graduação em Ecologia, Instituto de Biologia, Universidade Estadual de Campinas (UNICAMP), CP 6109, CEP 13083–970, Campinas, São Paulo, Brazil; 2 Departamento de Biologia Vegetal, Instituto de Biologia, Universidade Estadual de Campinas (UNICAMP), CP 6109, CEP 13083–970, Campinas, São Paulo, Brazil; 3 Departamento de Biologia Animal, Instituto de Biologia, Universidade Estadual de Campinas (UNICAMP), CP 6109, CEP 13083–970, Campinas, São Paulo, Brazil; University of Vienna, AUSTRIA

## Abstract

Predator activities may lead to the accumulation of nutrients in specific areas of terrestrial habitats where they dispose of prey carcasses. In their feeding sites, predators may increase nutrient availability in the soil and favor plant nutrition and growth. However, the translocation of nutrients from one habitat to another may depend on predator identity and diet, as well as on the amount of prey intake. Here we used isotopic (^15^N) and physiological methods in greenhouse experiments to evaluate the effects of the identity of predatory ants (i.e., the consumption of prey and nest sites) on the nutrition and growth of the bromeliad *Quesnelia arvensis*. We showed that predatory ants with protein-based nutrition (i.e., *Odontomachus hastatus*, *Gnamptogenys moelleri*) improved the performance of their host bromeliads (i.e., increased foliar N, production of soluble proteins and growth). On the other hand, the contribution of *Camponotus crassus* for the nutritional status of bromeliads did not differ from bromeliads without ants, possibly because this ant does not have arthropod prey as a preferred food source. Our results show, for the first time, that predatory ants can translocate nutrients from one habitat to another within forests, accumulating nutrients in their feeding sites that become available to bromeliads. Additionally, we highlight that ant contribution to plant nutrition may depend on predator identity and its dietary requirements. Nest debris may be especially important for epiphytic and terrestrial bromeliads in nutrient-poor environments.

## Introduction

Ecological research has increasingly focused on the links between species interactions and ecosystem functioning [1, 2]. Predators have a significant influence on ecosystems by affecting prey populations, and controlling the dynamics of nutrients, either by cascading effects through herbivore-plant-decomposers or directly through their excreta and activity of nutrient translocation [2]. As predators capture prey and transport them to their feeding sites, they may redistribute nutrients between habitats [3, 4]. Nutrient translocation by predators has been observed in riparian zones by bears that release marine and freshwater nutrients through deposition of salmon carcasses, raising ammonium and nitrate concentrations in the soil [3]. Similarly, seabirds and sea turtles feeding on marine organisms may excrete nutrients or leave egg remains in their breeding nests and nourish nearby soils [5, 6]. In addition, crows that feed in urban areas transfer nutrients to their roosts in adjacent forests [7]. These translocated nutrients may become available for plants and impact their performance and community diversity and structure [4].

The identity of predators and their hunting and feeding modes play a key functional role within communities and can determine the amount of nutrients that are translocated to soils [2, 8]. However, little is known about how predator identity and hunting mode can affect the nutrition of plants. In the absence of predators, some plants are able to capture or shelter a diversity of species that are not used by them as a direct source of nutrients. For example, the carnivorous plant *Roridula* captures prey but lack digestive enzymes, and depends on the activity of predatory hemipterans for prey digestion and release of nutrients [9]. Similarly, Romero *et al*. [10, 11] showed that while predatory spiders and frogs provide nutrients to their host bromeliads, entire prey carcasses contribute less to plant nutrition. Predatory ants may also contribute to plant nutrition, and many plants have modified hollow structures (i.e., domatia) that provide shelter to ant colonies, establishing symbiotic relationships [12–16]. Because ants have hunting sites often distinct from their feeding sites (i.e., colonies), ants can translocate nutrients from one habitat to another, similarly to bears, seabirds, sea turtles and the crows.

Symbiotic associations between plants and ants have been described for many species. Some plants are myrmecophytes (i.e., specialized ant-plants) that have modified hollow cavities (i.e., domatia) that house ant colonies in an obligate association, while other plants are myrmecophilous in a non-specialized association with ants [13–19]. In both cases, nest debris with food remains and ant feces are stored in contact with plant tissue, and nutrients can be obtained by plants [13–16]; ant-assisted plant nutrition is called myrmecotrophy. In the Neotropics, bromeliads are among the most used plants by ants [17, 18]. Some myrmecophytic bromeliads have bulb-shaped leaves that house ant colonies (e.g., *Brocchinia acuminata*, *Tillandsia bulbosa*, *T*. *butzii*), while others have ant nests constructed among roots or in inter-foliar cavities that do not accumulate water [18–21]. Tank bromeliads shelter many aquatic and terrestrial organisms, from microorganisms to vertebrates and retain leaves from the forest canopy [22, 23]. Therefore, tank bromeliads can be considered a complete ecosystem with debris, decomposers, detritivores, omnivores and predators [1]. Because they possess foliar trichomes capable of absorbing water and nutrients, bromeliads can obtain nutrients from the trophic network they shelter [10, 11, 22, 24]. Many ants that occupy bromeliads are predators that forage in the vicinity of their host plants and bring arthropod prey into their colonies. Thus, nest debris can be a source of nutrients for bromeliads, as Leroy *et al*. [15, 16] suggested by comparing leaf nitrogen isotopes between bromeliads with and without ant colonies.

In the nutrient-poor soils of coastal Atlantic Forests [25], the bromeliad *Quesnelia arvensis* hosts colonies of *Odontomachus hastatus*, *Gnamptogenys moelleri*, *Camponotus crassus* and two other ant species [26]. Among the ants that interact with this plant, nests of *O*. *hastatus* are located mainly among bromeliad roots [21, 27, 28]. On the other hand, *G*. *moelleri* and *C*. *crassus* have their nests among bromeliad leaves ([29, 30, 31, 32], AZG personal observations). While *O*. *hastatus* and *G*. *moelleri* have a predatory habit and feed mainly on arthropods, *C*. *crassus* feeds mostly on extrafloral nectaries, homopteran exudates, and fruits, but may also hunt for arthropods to supplement its diet [30–32]. As *O*. *hastatus*, *G*. *moelleri* and *C*. *crassus* capture and bring prey into their nests among bromeliad roots (*O*. *hastatus*) or bromeliad leaves (*G*. *moelleri* and *C*. *crassus*), they may translocate nutrients from one environment to another and improve plant nutrition, as shown for other predators [3–7]. Here we report the results of an experiment designed to evaluate the effects of the identity of ants and their diet (i.e., the consumption of prey and nest sites) on the nutrition and development of their host bromeliads. Assuming that these three ant species have nests with similar number of ants (see Methods), we hypothesized that *O*. *hastatus* and *G*. *moelleri* will contribute more to the nutrition and growth of *Q*. *arvensis*, since they have a predatory habit and their diet is based mainly on arthropods. On the other hand, we expect that *C*. *crassus* will contribute less to the nutrition and growth of host bromeliads because its diet is based on fewer prey items.

## Methods

### Ethics Statement

This study was conducted according to relevant national and international guidelines. Permit number 12.429/2011 for Ana Z. Gonçalves, issued by the *Secretaria do Meio Ambiente* and *Instituto Florestal*, in accordance with the *Instituto Brasileiro do Meio Ambiente e dos Recursos Naturais Renováveis (IBAMA)* and *Ministério do Meio Ambiente (ICMBio MMA)*.

### Organisms and field sampling

Colonies of *Odontomachus hastatus*, *Gnamptogenys moelleri* and *Camponotus crassus* were collected in the restinga forest of the Parque Estadual da Ilha do Cardoso (Cardoso Island), São Paulo State, Brazil (25°04’ S, 47°55’ W). The understory of the restinga is covered mostly by *Quesnelia arvensis*, whereas *Vriesea procera* is the most common epiphytic species [21, 23]. Nests of *O*. *hastatus* are frequently constructed in *V*. *procera*, but *Q*. *arvensis* can also host colonies [21]. *Quesnelia arvensis* is a tank bromeliad that obtains nutrient not only by its roots, but also by foliar trichomes that are capable of absorbing nitrogen compounds (e.g., amino acids) [22]. Among the ants that interact with *Q*. *arvensis*, *O*. *hastatus* has the greatest size (≈ 1.3 cm) and biomass (mean ± SE, 7.89 ± 1.57 mg) and their nests are abundant (ca. 33 colonies. ha^-1^) among bromeliad roots [21, 27, 28]. This nocturnal ant species is arboreal and occurs from Central to South America [33]; its nests are rarely found on the ground [27, 34]. Rodrigues and Oliveira [28] showed that *O*. *hastatus* workers can move more than 8 m away from their nests, but nearly half of the foraging activity occurs within 3 m around the nest. *Gnamptogenys moelleri* has ≈ 0.5 cm in length, 1.15 ± 0.32 mg in biomass, occurs in Neotropical plains, its nests can be found on the ground and in terrestrial bromeliads among 1–3 leaves, and workers forage almost exclusively on their host plants [29, 30]. *Camponotus crassus* (≈ 0.5 cm in length, 1.04 ± 0.09 mg in biomass) nests among bromeliad leaves and feeds mostly on extrafloral nectaries, homopteran exudates and fruits, and can suppress herbivores from plants that have extrafloral nectaries ([31, 32, 35], AZG personal observations). On Cardoso Island, nests of this ant are found among 2–3 leaves of *Q*. *arvensis*, where there is no water accumulation (AZG personal observations).

The number of ants per colony did not differ among treatments of the experiment described below (One-way ANOVA, P = 0.261; mean ± SE, 129.3 ± 7.6 ants *O*. *hastatus* colonies; 141.7 ± 4.7 ants in *G*. *moelleri* colonies; and 149.0 ± 11.4 ants in *C*. *crassus* colonies). However, the nest biomass differed among species (One-way ANOVA, P < 0.001; mean ± SE, 1020.1 ± 60.1 mg of *O*. *hastatus*; 162.9 ± 5.5 mg of *G*. *moelleri*; and 154.9 ± 11.8 mg of *C*. *crassus*).

### Greenhouse experiment

Previously to the experiment, *Tenebrio* larvae (simulating prey of ants) were enriched with ^15^N stable isotope to quantify the flux of nitrogen from *Tenebrio* to bromeliads. *Tenebrio* were grown in a substrate in the proportion of 100 g of rat chow Labina-Purina®, 100 g cassava flour and 20 mL of a solution of 5 g enriched ammonium sulfate [(^15^NH_4_)_2_SO_4_, 10% excess atoms, Cambridge Isotope Laboratories, MA] per liter of distilled water. This substrate was dried for 24 h at 60°C before being offered to *Tenebrio*.

In order to test whether ants alter the availability of nutrients to their host bromeliads due to prey intake and nest sites, a two-month (10-Jan-2012 to 18-Mar-2012) greenhouse experiment was carried out at the Department of Plant Biology at Universidade Estadual de Campinas, Brazil. Since *O*. *hastatus* can be found in *Q*. *arvensis* [21], this species was chosen for the experiment and was obtained at Veiga Arquitetura e Paisagismo®, CEASA, Campinas, Brazil. All bromeliads were young and had similar biomass and size (e.g., foliar length varying from 25 to 30 cm). Bromeliads were planted in pots (14.5 cm in diameter, 14.5 cm high) with *Pinus* sp. bark (simulating the poor soil of restinga forest; Martins *et al*. 2015) and were watered with limited amounts of water to avoid desiccation through an automatic irrigation system with a capacity of 8L.h^-1^, which worked for 10 min every 2 h. Each pot was kept individually in a white plastic tray (40.7 x 60.8 x 9.8 cm) with Tanglefoot® resin around its border to prevent ants leaving their host bromeliads. All pots had four holes at their bases (approximately 1 x 1 cm) to allow ants to access the bromeliad roots. Colonies of *O*. *hastatus* collected in the field were placed in the plastic trays and quickly sought refuge and entered the holes of the pots in which bromeliads were planted. In about 30 min, all ants entered the pots and carried eggs and pupae to establish the colony within the roots. Colonies of *G*. *moelleri* and *C*. *crassus* were manually placed in the rosette of potted bromeliads, protecting eggs and pupae with *Pinus* sp. barks (the same used inside pots among bromeliad roots).

Experimental bromeliads had the following treatments (*n* = 10 each treatment): (1) control; (2) no ants but with *Tenebrio* larvae placed among roots (to test the amount of nitrogen that bromeliads acquire through roots); (3) no ants but with *Tenebrio* placed on leaves (to test the amount of nitrogen derived from insects that fall into the rosettes); (4) with *C*. *crassus* colony and *Tenebrio* placed on leaves (to test if ants accelerate the acquisition of nitrogen through leaves); (5) with *G*. *moelleri* colony and *Tenebrio* placed on leaves (to test if ants accelerate the acquisition of nitrogen through leaves); and (6) with *O*. *hastatus* colony among roots and *Tenebrio* placed on leaves (to test if ants accelerate the acquisition of nitrogen through roots). Two *Tenebrio* larvae were cut into six pieces and were applied every other day on each bromeliad (except on control plants), in the center of the rosette above the tank in treatments *3*, *4*, *5 and 6*. *Tenebrio* was the only nutrient source for the colonies, and ants were very fast at collecting *Tenebrio* on leaves. *Tenebrio* was placed among roots (treatment *2*) through a PVC pipe (2 cm in diameter) inserted in the root mass. At the end of the experiment, two leaves of the second inner node of each bromeliad rosette were collected for isotope analyses, and three leaves of the fifth inner node of the rosettes were collected for soluble protein analyses.

### Analyses of plant protein

Total soluble protein concentration was determined using the colorimetric Bradford assay [36]. Fresh leaves of *Q*. *arvensis* were cut into small pieces (1 cm^2^), and 1 g of these leaves was frozen in liquid nitrogen and homogenized with 3 mL of ultra-pure water. The homogenate was centrifuged at 12.000 rpm (g) for 10 min, and the supernatant (15 μL) was mixed with Comassie Brilliant Blue G-250 dye solution (185 μL), obtained from 100 mg of the dye dissolved in 95% ethanol and 100 mL of 85% phosphoric acid. The absorbance was measured using a spectrophotometer (Ultrospec 3000; Cambridge, England) at 595 nm, and the concentration of protein was determined by plotting the absorbance of the sample *vs*. a standard curve obtained with bovine serum albumin.

### Bromeliad growth

To determine the relative contribution of nitrogen derived from *Tenebrio* only or from ant nests on bromeliad growth, two leaves (i.e., fifth inner node of the rosettes) from each bromeliad were randomly chosen and their lengths were measured at the beginning and the end of the experiment. The bromeliad leaf length was directly related to the leaf biomass (Linear regressions: *Q*. *arvensis*: *r*^2^ = 0.73, P < 0.001); leaves showed continuous growth during the experiment and their relative growth rate (RGR, Ln(cm)/day) was calculated using the following equation: RGR = [ln(L_final_)–ln(L_initial_)]/(t_2_ − t_1_). The ln(L_final_) and ln(L_initial_) are, respectively, the natural logarithm of the foliar final length and the natural logarithm of the foliar initial length, with (t_2_ –t_1_) being the number of days between the initial and final measurements.

### Isotopic and statistical analyses

The total N concentration (μg mg^-1^ dry leaf tissue) of bromeliad leaves and the δ^15^N of *Tenebrio*, ants and bromeliads were determined with an isotope ratio mass spectrometer (20–20 mass spectrometer; PDZ Europa, Sandbach, UK) after sample combustion to N_2_ at 1000°C by an on-line elemental analyzer (PDZ Europa ANCA-GSL) in the Stable Isotope Facility at the University of California, Davis. The nitrogen fraction in bromeliads with and without ants that received *Tenebrio* (f_A_) were calculated using mixing model equations with two sources of nitrogen (*i*.*e*., soil and *Tenebrio*) and one single isotopic signature (*e*.*g*., δ^15^N; see [37]). Since control bromeliads only had *Pinus* sp. bark substratum as a source of nutrients, leaves of these bromeliads were considered the soil end-member in the equation. The fractionation of ^15^N during its assimilation and metabolic processing in plants [38] was considered in the following equation: f_A_ = (δ_M_ − δ_B_ − Δδ^15^N)/(δ_A_ − δ_B_). The f_A_ is the proportionate contribution of labelled *Tenebrio* absorbed by bromeliads (%), δ_M_ is the isotope ratio of bromeliads that received *Tenebrio*, δ_A_ is the isotope ratio of *Tenebrio* while δ_B_ is the isotope ratio of control bromeliads, and Δδ^15^N is the trophic shift for nitrogen between *Tenebrio* or control bromeliads and consumer (*e*.*g*., bromeliads). The values of Δδ^15^N used were + 1.4 ± 0.21‰ (mean ± SE) [39]. All the response variables were compared using ANOVA and Tukey HSD *post-hoc* test were used for pair-wise comparisons.

## Results

**Fig 1:**
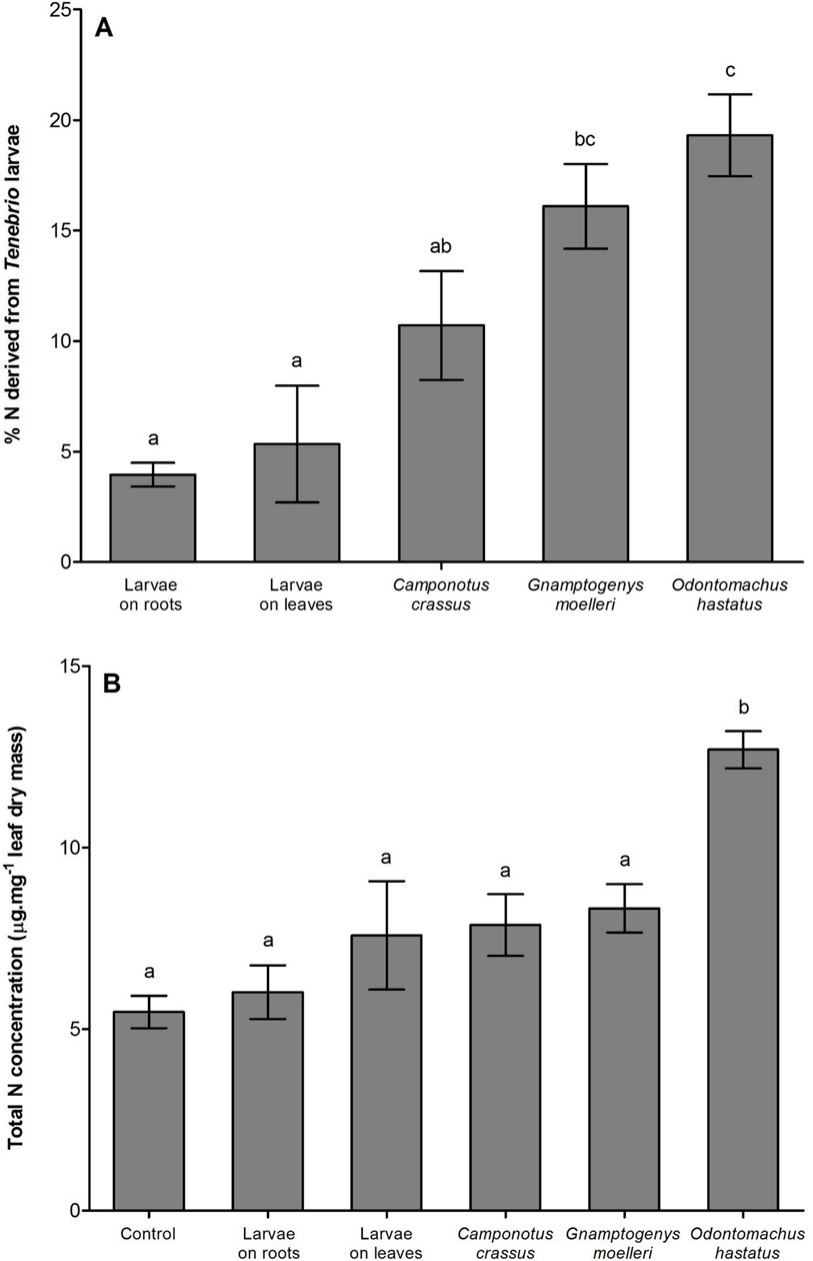
Percentage of nitrogen derived from prey and total nitrogen concentration of bromeliads. (A) Percentage of nitrogen derived from *Tenebrio* larvae and (B) total nitrogen concentration of *Quesnelia arvensis* leaves of different treatments (control; *Tenebrio* among roots; *Tenebrio* on leaves; *Camponotus crassus* ants and *Tenebrio*; *Gnamptogenys moelleri* and *Tenebrio*; and *Odontomachus hastatus* and *Tenebrio*). Bars indicate the standard error and letters indicate Tukey *post-hoc* test (α < 0.05).

**Fig 2:**
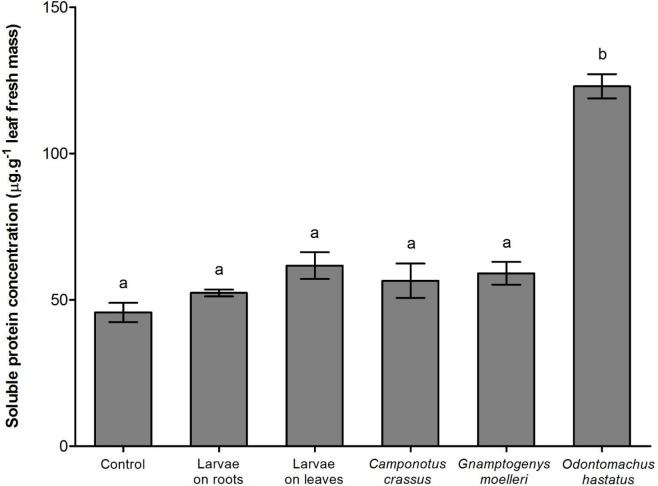
Soluble protein concentration of bromeliads. Soluble protein concentration for *Quesnelia arvensis* leaves of different treatments (control; *Tenebrio* larvae among roots; *Tenebrio* on leaves; *Camponotus crassus* ants and *Tenebrio*; *Gnamptogenys moelleri* and *Tenebrio*; and *Odontomachus hastatus* and *Tenebrio*). Bars indicate the standard error and letters indicate Tukey *post-hoc* test (α < 0.05).

**Fig 3:**
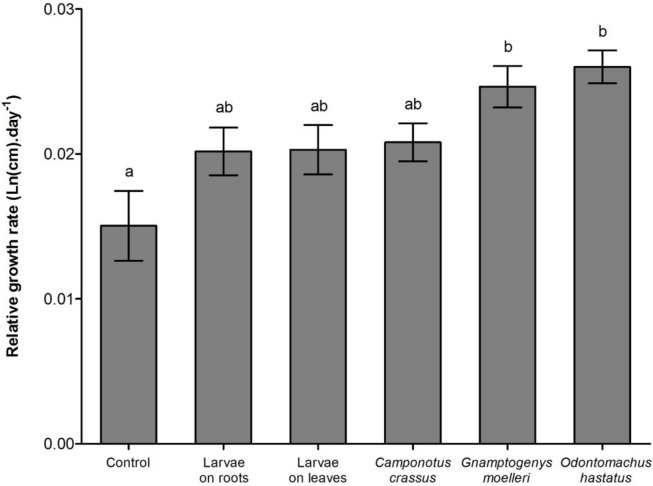
Relative growth rate of bromeliads. Relative growth rate of the *Quesnelia arvensis* leaves from different treatments (control; *Tenebrio* larvae among roots; *Tenebrio* on leaves; *Camponotus crassus* ants and *Tenebrio*; *Gnamptogenys moelleri* and *Tenebrio*; and *Odontomachus hastatus* and *Tenebrio*). Bars indicate the standard error and letters indicate Tukey *post-hoc* test (α < 0.05).

The δ^15^N values of enriched *Tenebrio* larvae, of the ants *Odontomachus hastatus*, *Gnamptogenys moelleri* and *Camponotus crassus* that fed on the enriched *Tenebrio* indicate that these materials were enriched during the experiment (Table 1). As expected, *Odontomachus hastatus* contributed more to the nutrition of its host bromeliad, accounting for 19.3 ± 1.8% (mean ± SE) of the total nitrogen of *Quesnelia arvensis* (Tables 1 and 2; Fig 1A). *Gnamptogenys moelleri* contributed 16.1 ± 1.9% of the nitrogen of its host bromeliad, whereas *C*. *crassus* contributed with 10.7 ± 2.4% (Tables 1 and 2; Fig 1A). In the absence of ants, *Tenebrio* on leaves contributed with 5.3 ± 2.6% of the nitrogen of bromeliads, and *Tenebrio* on roots contributed with 3.9 ± 0.5% (Tables 1 and 2; Fig 1A). Despite differences among treatments in the contribution to bromeliad nutrition, only *O*. *hastatus* contributed to the increase of total nitrogen and soluble protein concentrations in bromeliad leaves (Table 2; Figs 1B and 2). Bromeliads with *G*. *moelleri* and *C*. *crassus* colonies had total nitrogen and soluble protein concentrations similar to bromeliads with prey carcasses or control bromeliads (Table 2; Figs 1B and 2). Additionally, *O*. *hastatus* and *G*. *moelleri* improved foliar growth of their host plants compared to control bromeliads while *C*. *crassus* had no effect on bromeliad growth (Table 2; Fig 3).

**Table 1:** δ^15^N values. Average δ^15^N values of natural abundance and enriched *Tenebrio* larvae, *Camponotus crassus*, *Gnamptogenys moelleri* and *Odontomachus hastatus* ants, and *Quesnelia arvensis* leaves receiving the following treatments: control; *Tenebrio* among roots; *Tenebrio* on leaves; *Camponotus crassus* ants and *Tenebrio*; *Gnamptogenys moelleri* and *Tenebrio*; and *Odontomachus hastatus* and *Tenebrio*.

*Treatment*	δ^15^N values (SE)	N
*Tenebrio*		
Natural abundance	3.13 (0.12)	5
Enriched	76156.0 (2940.62)	5
*Camponotus crassus*		
Natural abundance	0.8 (1.06)	5
Enriched	36713.5 (392.19)	5
*Gnamptogenys moelleri*		
Natural abundance	2.6 (0.37)	5
Enriched	47087.9 (714.54)	5
*Odontomachus hastatus*		
Natural abundance	2.4 (0.41)	5
Enriched	55959.6 (1208.23)	5
Control		
Natural abundance	6.33 (0.60)	10
*Tenebrio* on roots		
Enriched	3025.0 (371.28)	10
*Tenebrio* on leaves		
Enriched	4079.6 (1902.84)	10
Bromeliad with *C*. *crassus*		
Enriched	8167.8 (1748.73)	10
Bromeliad with *G*. *moelleri*		
Enriched	12272.8 (1292.63)	10
Bromeliad with *O*. *hastatus*		
Enriched	14715.3 (1194.74)	10

The standard errors of means are in parenthesis.

N, Number of replicates.

**Table 2:** % of N, total N and soluble protein concentrations, and relative growth rate of bromeliads. Analyses of variance (ANOVA) summarizing the effects of different treatments (control; *Tenebrio* larvae among roots; *Tenebrio* on leaves; *Camponotus crassus* ants and *Tenebrio*; *Gnamptogenys moelleri* and *Tenebrio*; and *Odontomachus hastatus* and *Tenebrio*) on the % of N derived from *Tenebrio*, the total N and soluble protein concentrations, and relative growth rate of *Quesnelia arvensis* leaves. Significance of *P < 0*.*05* is highlighted in bold.

Source of variation	d.f.	MS	*F*	*P*
% N derived from *Tenebrio*				
Treatments	4	441.80	10.84	**<0.001**
Error	45	40.77		
Total N concentration				
Treatments	5	65.38	8.91	**<0.001**
Error	54	7.33		
Soluble protein concentration				
Treatments	5	8007.70	48.04	**<0.001**
Error	54	167.70		
Relative growth rate				
Treatments	5	0.00	5.46	**<0.001**
Error	54	0.00		

## Discussion

In this study, we assessed how the identity and diet of ant inhabitants can affect the nutrition of host plants through the translocation of prey carcasses and feces by predators. We found that the ant species with protein as their main food source (i.e., *Odontomachus hastatus*, *Gnamptogenys moelleri*) had a higher contribution to the performance of their host bromeliads (i.e., plant nutrition, production of soluble proteins, and growth). Regardless of the ant species, nitrogen physically stored in the body of prey becomes available to bromeliads.

We demonstrate experimentally that predatory ants play a role on bromeliad nutrition since they concentrate prey carcasses in their feeding sites (i.e., colonies in bromeliads) and release nutrients from prey bodies through nest debris and feces. We also demonstrate that predators that have arthropod prey as their main source of protein contribute more to the bromeliad performance (i.e., *O*. *hastatus* and *G*. *moelleri*). Although *G*. *moelleri* and *O*. *hastatus* have similar predatory habit [27, 30], the former species showed an intermediate contribution to its host plant nutrition, possibly due to its small biomass (similar to *C*. *crassus*). These results reinforce that the dietary requirements of each species must be taken into account to evaluate the potential nutrient-enrichment by predators in a community [8].

*Tenebrio* larvae (simulating prey that fall into the tank of bromeliads) contributed less to plant nutrition than the presence of predatory ants on bromeliads. This result emphasizes the role of ants in processing the organic matter of prey bodies, releasing compounds through the nest debris and their feces, which can be obtained by bromeliad roots (e.g., when plant interact with *O*. *hastatus*) or by leaf trichomes (e.g., when interacting with *G*. *moelleri* or *C*. *crassus*). Other studies also showed that predator-plant interactions could release organic matter from prey bodies retained by plants [10, 40, 41, 42]. In the absence of predators, *Tenebrio* need to be mineralized by microorganisms present in the bromeliad rosettes [24, 43], a process that should be slower than predator activity, which could explain the lower contribution of *Tenebrio* to bromeliad nutrition during the experiment.

The terrestrial bromeliad *Quesnelia arvensis* is very abundant in the sandy nutrient-poor soils of Cardoso Island [23, 25]. Through the interaction with predators, this plant can obtain nutrients from the soil via its roots or leaf trichomes that are in contact with debris and feces from ant nests. In fact, we showed that the interaction between *Q*. *arvensis* and ants favored plant nutrition and enabled a higher production of soluble protein in its leaves. High soluble protein concentration is associated with the N availability in plants indicating their favorable nutritional status [44], which may have allowed a higher growth of *Q*. *arvensis* when interacting with ants. In addition, the increase in N concentration in leaves of bromeliads can favor the production of amino acids usually associated with N storage [45], and can be especially important for epiphytes whose roots are not in contact with soil and have intermittent access to water and nutrients. At Cardoso Island, nearly 70% of the *O*. *hastatus* nests were recorded in the epiphytic *Vriesea procera* [27]. Therefore, it is expected that *V*. *procera* can benefit even more from interactions with ants than the terrestrial *Q*. *arvensis*.

The contribution of *O*. *hastatus* and *G*. *moelleri* to bromeliads were similar to the N values that the carnivorous plants *Cephalotus*, *Drosera*, *Philcoxia* and *Sarracenia* derived from insect digestion [46–48]. Despite the existence of carnivorous bromeliads (i.e., *Catopsis*, *Brocchinia*), *Q*. *arvensis* is considered a saprophytic species because it lacks an active way to attract, immobilize and digest prey [49]. The interactions described here represent a by-product digestive mutualism between ants and bromeliads where ants can find a suitable habitat in bromeliads and, in turn, contribute nutritionally to their hosts [10, 11, 40]. Finally, some researchers have argued that ants preferably associate with bromeliads with longer leaves and larger rosettes and root mass [21, 30]. We suggest that the larger size of ant-inhabited bromeliads could be a result of this by-product mutualism, and not necessarily the cause mediating plant colonization by ants.

In conclusion, our results emphasize the effects of predator identity and diet in the translocation and processing of prey nutrients that contribute to plant nutrition. Predatory ants with that feed on prey as their main source of protein contributed more to the nutrition, protein production and growth of their host bromeliads. These results emphasize that ants can play a role in redistributing nutrients between habitats, from different areas of the forest to their feeding sites (i.e., colonies). We highlight the importance of predator activities in concentrating wastes near their feeding sites and their potential to provide nutrients to plants, and we also reinforce that the by-product digestive mutualism can be relevant to plants that occur in oligotrophic environments.
